# Potential Anti‐Aging Effects of a Dietary Supplement From the Algal‐Derived Omega‐3 DHA in Aged SAMP 8 Mice

**DOI:** 10.1002/fsn3.71353

**Published:** 2026-01-30

**Authors:** Ming‐Yu Chou, I‐Hung Lin, Chia‐Jung Chen, Ting‐Jian Guo, Che‐An Lin, Dao‐Na Yang, Po‐Hsien Li, Yu‐Chen Li, Mei‐Due Yang, Chieh‐Chang Tien, Ruei‐Ze Lin, Ching‐Hsin Chi, Shih‐Yi Wang, Ming‐Fu Wang

**Affiliations:** ^1^ Wenzhou Medical University Big One Health Development Research Institute Wenzhou City Zhejiang Province China; ^2^ International Aging Industry Research & Development Center (AIC), Providence University Taichung Taiwan; ^3^ Y.C.C. Parts MFG Co., Ltd. Lukang Township Changhua County Taiwan; ^4^ Department of Food and Nutrition Providence University Taichung Taiwan; ^5^ Department of Surgery, Department of Clinical Nutrition China Medical University Hospital Taichung Taiwan

**Keywords:** algal‐derived omega 3‐DHA, antiaging, antioxidant, learning and memory ability, senescence‐accelerated mouse prone‐8 (SAMP8)

## Abstract

This study investigated the anti‐aging effects of algal Omega3‐DHA in senescence‐accelerated mice (SAM). Male and female SAMP8 mice (3 months old) were divided into a control group and four experimental groups (1× or 2× algal Omega3‐DHA, with/without phospholipids). The mice were orally administered test samples dissolved in corn oil daily for 13 weeks. Aging scores were significantly lower in male mice across all experimental groups and in female mice in the phosphatidylcholine (PC) and phosphatidylserine (PS) (*p* < 0.05) groups. Learning and memory improved significantly in all the experimental groups (*p* < 0.05). Brain biomarkers of aging, including 8‐hydroxy‐2′‐deoxyguanosine (8‐OHdG), thiobarbituric acid‐reactive substances (TBARS), protein carbonyl content, and β‐amyloid (Aβ) protein, were significantly reduced, while liver antioxidant enzyme activities, including superoxide dismutase (SOD), catalase (CAT), and glutathione peroxidase (GPx), were increased in the PC and PS groups (*p* < 0.05). Additionally, survival times were extended in both male and female mice compared to controls. No adverse effects were observed in terms of body weight or activity level. In summary, algal Omega3‐DHA supplementation improved cognitive performance, enhanced antioxidant defenses, reduced aging markers, and delayed aging in SAM mice, highlighting its potential as a promising anti‐aging strategy.

## Graphical abstract

Algal‐derived Omega‐3 DHA supplementation exerts significant anti‐aging and neuroprotective effects in SAMP8 mice. Algal Omega‐3 DHA effectively mitigates age‐related cognitive decline, enhances antioxidant defenses, and extends lifespan. Algal‐derived Omega‐3 DHA supplementation enhances cognitive function, attenuates oxidative stress, and reduces neurodegenerative markers.

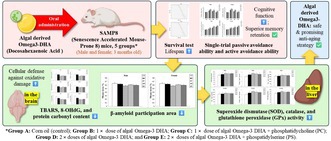

## 1 Introduction

According to the World Health Organization (WHO), the number and proportion of people aged 60 years and older in the population is increasing. In 2021, the number of people aged 65 years was 761 million. This number will increase to 1.4 billion by 2030 and 2.1 billion by 2050. Moreover, the last decade of life is often accompanied by disease, with cognitive impairment and dementia being important contributing factors. Patients with dementia gradually lose their ability to care for themselves, accompanied by a worsening nutritional status, becoming an increasingly serious public health issue (Mohajeri et al. 2015).

Alzheimer's disease (Anthuparambil et al. 2023) is a common neurodegenerative disorder characterized by progressive cognitive decline. A central pathological hallmark of AD is the abnormal metabolism of amyloid precursor protein (APP), leading to the accumulation of Aβ plaques in the brain (Rahmati et al. 2023). APP can be processed via two pathways: the non‐amyloidogenic pathway, which precludes Aβ formation, and the amyloidogenic pathway, which generates Aβ peptides, particularly Aβ40 and Aβ42 (Sun et al. 2015). These peptides aggregate into oligomers and fibrils, forming extracellular amyloid plaques that disrupt neuronal function, cause synaptic loss, and trigger neuroinflammation (Liu et al. 2024). Aggregation of Aβ peptides into amyloid plaques is a hallmark of AD pathology. These plaques disrupt neuronal function and are associated with synaptic loss and neuroinflammation (Liu et al. 2024). Additionally, Aβ deposition has been linked to blood–brain barrier (BBB) dysfunction, which exacerbates the accumulation of neurotoxic Aβ and contributes to disease progression (Wang et al. 2021). Furthermore, the interaction between Aβ and tau proteins plays a significant role in AD pathogenesis, with initial levels of Aβ deposition influencing the accumulation of tau tangles, which are another key feature of the disease (Cai et al. 2023). These insights underscore the complexity of Aβ deposition in AD and highlight the importance of targeting multiple pathological processes in therapeutic strategies.

Docosahexaenoic acid (DHA), a long‐chain omega‐3 polyunsaturated fatty acid, plays a crucial role in brain function, neuroprotection, and aging‐related metabolic regulation. It is an essential structural component of neuronal membranes, contributing to synaptic plasticity, neurotransmission, and cognitive function (Dyall 2015). DHA can promote anti‐inflammatory responses (Serhan and Levy 2018), and its antioxidant capacity can reduce oxidative stress in the brain (Faccinetto‐Beltrán et al. 2024), thereby reducing the risk of developing AD. DHA is primarily derived from marine sources, including fish oil and microalgae.

Recent studies suggest that the bioavailability and biological activity of DHA strongly depend on its molecular form. Traditional fish oil‐derived DHA is primarily present as triglycerides (DHA‐TAG) or ethyl esters (DHA‐EE), while DHA derived from marine phospholipids (DHA‐PL), such as DHA‐phosphatidylcholine (DHA‐PC) and DHA‐phosphatidylserine (DHA‐PS), exhibits higher absorption efficiency and greater brain uptake due to its superior ability to cross the BBB (Zhao et al. 2020). DHA‐PC and DHA‐PS are major components of biological membranes. PC supports the membrane structure, while PS promotes the growth and differentiation of neural cells, both of which possess neuroprotective and antioxidant properties (Hashioka et al. 2007).

Aging is closely associated with oxidative stress, mitochondrial dysfunction, and chronic neuroinflammation, which contribute to cognitive decline, Aβ accumulation, and neurodegenerative diseases such as Alzheimer's and Parkinson's disease (Calder 2015). Emerging evidence suggests that plant‐based DHA supplementation, particularly in DHA‐PL forms, may enhance its protective effects against oxidative damage and neuronal aging. Unlike conventional DHA supplements, plant‐based DHA contains naturally occurring bioactive compounds such as polyphenols, flavonoids, and carotenoids, which work synergistically to reduce oxidative stress and neuroinflammation (Gutiérrez et al. 2019). These phytochemicals may provide additional benefits in protecting neuronal integrity, stabilizing mitochondrial function, and regulating inflammatory pathways (e.g., IL‐1β and TNF‐α), thereby further reinforcing the neuroprotective and antiaging effects of DHA. Studies on aging models, such as SAMP8 mice, have demonstrated that DHA can delay age‐related cognitive decline, enhance antioxidant enzyme activity (e.g., SOD and GPx), and inhibit tau protein hyperphosphorylation and Aβ aggregation. Another study found that dietary supplementation with DHA‐PC and DHA‐PS improved the brain lipid profiles in SAMP8 mice fed a high‐fat diet (Zhao et al. 2020). This includes the recovery of essential phospholipids, such as DHA‐PS and ethanolamine plasmalogen, which are critical for maintaining cognitive function.

The recommended daily intake of Omega‐3 polyunsaturated fatty acids (Omega‐3 PUFA), primarily obtained from marine animals, is increasingly being met with algal‐based sources of Omega‐3 PUFA because of the depletion of marine resources and the bioaccumulation of toxic substances in animal fat tissues (accumulation of methylmercury in large fish and accumulation of microplastics in smaller fish) (Pennino et al. 2020). However, growing concerns over marine pollution (e.g., heavy metals, polychlorinated biphenyls, and microplastics) and sustainability issues have led to increasing interest in plant‐based DHA as an alternative source (Minihane et al. 2015). Therefore, to ensure adequate nutritional intake for health needs and global sustainable development, an increasing number of people are using algal‐based sources of Omega‐3 PUFA (Rizzo et al. 2023).

Algal‐derived DHA, an Omega‐3 PUFA derived from algal‐based or algal sources such as *Schizochytrium* and *Crypthecodinium cohnii*, is recognized for its sustainable production and suitability for vegetarian and vegan diets (Karnwal and Mohammad Said Al‐Tawaha 2023). Algal‐derived DHA is vital for brain health, especially during the last trimester of pregnancy and the first 2 years of life, supporting neuronal growth and synaptogenesis (Bradbury 2011).

Adequate DHA levels are linked to improved cognitive performance, including memory and learning, by enhancing synaptic plasticity and neurotransmission (von Schacky 2021). Furthermore, DHA influences neurotransmitter pathways, potentially alleviating the symptoms of depression and anxiety by modulating serotonin and dopamine levels (Batten et al. 2024). Although alpha‐linolenic acid (ALA)‐rich oils, such as flaxseed oil, can serve as DHA sources, the human body's conversion of ALA to DHA is limited, with a conversion rate of less than 1% (Yuan et al. 2022). This underscores the importance of obtaining DHA directly from dietary sources and supplements. Research indicates that supplementing rodents with algal‐derived DHA increases the DHA content in plasma and red blood cells (van Wijk et al. 2016), while also demonstrating significant benefits in SAMP8 mice, particularly in enhancing cognitive function and antioxidant capacity (Vela et al. 2019). Although marine‐derived DHA has been extensively studied in the context of brain aging and neurodegenerative disorders, there is a pressing need to explore whether algal‐derived DHA, particularly when combined with phospholipids, can achieve similar or superior bioavailability and neuroprotective effects.

This study aims to investigate the effects of algal‐derived DHA supplementation on oxidative stress reduction, cognitive preservation, and lifespan extension in aging SAMP8 mice. Specifically, it seeks to evaluate the antiaging efficacy, safety, and survival outcomes of algal‐based DHA in the context of aging and neurodegeneration, positioning it as a promising and sustainable alternative to marine‐derived DHA for preventive healthcare and personalized nutrition.

## 2 Materials and Methods

### 2.1 The SAMP8 Mouse

SAMP8 animals are senescence‐accelerated strains that spontaneously develop from breeding pairs of the AKR/J series, which were developed by Tokyo University in Japan, resulting in an accelerated aging animal model. SAMP8 mice characterized by neuronal cell loss in the brain, cortical atrophy, lipofuscin, sponge‐like changes in the brainstem reticular formation, amyloid deposition, rapid aging of other organs, and a shortened lifespan, were used in this experiment. Therefore, they are suitable experimental animal models for research related to delayed aging. SAMP8 mice were housed in transparent plastic cages [30 (W) × 20  × 10 (H) cm^3^]. The temperature in the laboratory animal room was maintained at 22°C ± 2°C with a relative humidity of 65% ± 5%, and it was a dust‐free environment with automatic control. Lighting cycles were controlled using an automatic timer. From 07:00 to 19:00, this was the light period, while from 19:00 to 07:00, it was the dark period. Food and water were provided ad libitum, allowing animals to feed freely.

### 2.2 Sample Preparation

*Schizochytrium* sp. (ATCC 20888) was purchased from the American Type Culture Collection (ATCC). According to a study by Han et al. (2024), Schizochytrium was cultivated on solid glucose‐yeast‐peptone (GYP) medium and incubated at 28°C for 24 h in a rotary shaker set to 230 rpm. Subsequently, a 5% (vol/vol) inoculum was introduced and further cultured at 28°C for 120 h in a rotary shaker set to 250 rpm.

According to Ding et al. (2022), the fermentation broth was centrifuged, and the precipitate was washed twice with double‐distilled water, followed by another round of centrifugation and subsequent freeze‐drying. One gram of freeze‐dried powder was mixed with 8 mL of 6 mol/L hydrochloric acid and incubated in a hot water bath at 65°C for 1 h. Total fatty acids were extracted using 10 mL of n‐hexane, and the extraction process was repeated three times. n‐Hexane was then evaporated using a rotary nitrogen blower to collect total lipids. The extracts were subsequently formulated into capsules containing 1% phosphatidylcholine (PC) and phosphatidylserine (PS).

### 2.3 Survival Test

Dosage conversion was based on the evaluation methods specified by the Ministry of Health and Welfare. The dosage conversion was based on the human equivalent dose (HED) method using a scaling factor of 12.3, as recommended by the US FDA (2005) and adopted by the Ministry of Health and Welfare, Taiwan. The human recommended intake was 1200 mg/day for a 60 kg adult (i.e., 20 mg/kg BW/day), resulting in mouse‐equivalent doses of 123 mg/kg BW/day (0.5×), 246 mg/kg BW/day (1×), and 492 mg/kg BW/day (2×). The tests were conducted using 6‐month‐old SAMP8 mice, which were randomly assigned into four groups (*n* = 20 males and 20 females per group). The control group received corn oil, while the experimental groups were administered algal‐derived DHA at doses of 123 mg/kg BW/day (0.5×), 246 mg/kg BW/day (1×), and 492 mg/kg BW/day (2×), respectively. All animals were gavaged once daily. The number of deaths was recorded daily. Survival curves were plotted to compare the mean and maximum lifespans across groups, and the median survival time (i.e., the number of days until 50% mortality) was calculated.

### 2.4 Experiment Design

**FIGURE 1:**
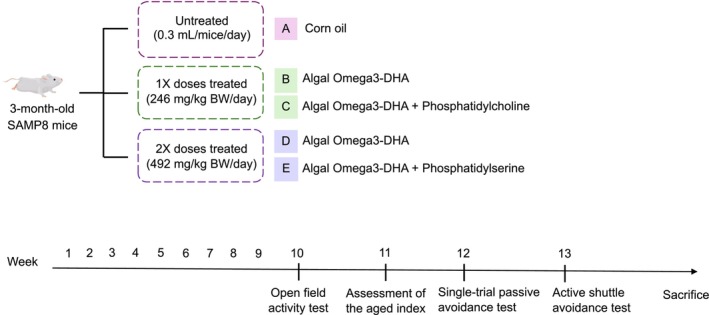
The experiment design.

3‐month‐old male and female SAMP8 mice were used as experimental animals. The experimental design is illustrated in Figure 1. A total of 120 male and female SAMP8 mice were used as experimental animals, with 24 mice (12 males and 12 females) assigned to each of the five treatment groups: corn oil as control group A, 1× doses of algal Omega‐3 DHA as group B, 1× doses of algal Omega‐3 DHA + PC as group C, 2× doses of algal Omega‐3 DHA as group D, and 2× doses of algal Omega‐3 DHA + PC as group E.

All test samples were freshly prepared each day. Each mouse received a once‐daily oral gavage using a stainless‐steel feeding needle, at a volume of 0.3 mL per mouse per day, for 13 consecutive weeks. In the 10th week, a locomotion test was conducted. In the 11th week, aging scores were assessed. In the 12th week, a single‐trial passive avoidance test was conducted, and in the 13th week, an active shuttle avoidance test was performed to evaluate learning and memory abilities. After the behavioral tests, the mice were euthanized by cardiac blood collection under carbon dioxide anesthesia (with an 8‐h fasting period prior to euthanasia). Blood and organs were collected for various data analyses. During the experiment, the body weight and food intake were measured daily, and water intake was recorded in milliliters per day.

### 2.5 Open Field Activity Test (Locomotion Activity)

In the 10th week of the experiment, a locomotion test was conducted. Mice were placed in the center of a 25 (W) × 25 × 25 (H) cm^3^ aluminum box (activity monitor video path analyzer, Coulbourn Instruments Model E61‐21). The test was conducted in a dimly lit, quiet environment. Each group of mice was subjected to a test. The entire session was recorded to observe the mice's horizontal movement time (locomotion; s/5 min). The activity was recorded every 5 min for a total duration of 10 min to understand the horizontal movement of the mice, serving as the basis for evaluating their activity levels.

### 2.6 Assessment of the Aged Index

The aging index assessment includes observing the behavior, appearance, eyes, and spine of mice. An exploratory response within 30 s and an escape response when pinching the nape skin were observed. Appearance assessment involves checking the glossiness, coarseness, hair loss of fur, and any skin ulcers. Eye assessment focuses on identifying mucosal inflammation or eyelid edema around the eye. Finally, spinal assessment involves inspecting and palpating the changes in the curvature. Each of these aspects is rated on a scale from 0 to 4, with higher scores indicating more severe aging.

### 2.7 Single‐Trial Passive Avoidance Test (PAT)

In this experiment, a 35 (W) × 17 × 20 (H) cm^3^ aluminum box (shuttle cage, Coulbourn Instruments Model E10‐15) was used, divided into a light chamber and a dark chamber separated by a small guillotine door (7.5 (W) × 6.5 cm^2^, Coulbourn Instruments Model E10‐15GD). The floor of the box had metal rods arranged in parallel at 1 cm intervals, connected to a current generator. During the test, a mouse was placed in the light chamber for a 10‐s acclimation period before the door was opened to allow free exploration. Owing to their nocturnal behavior, mice naturally moved toward the dark chamber. Once the mouse entered the dark chamber, the door closed quickly, and after 5 s, a 0.5‐s shock of 0.5 microamperes was administered, repeated three times at 5‐s intervals to complete the learning training. Memory was tested 24 and 48 h later using the same procedure but without any shocks. The time spent in the light chamber was recorded, with a maximum duration of 180 s; longer durations indicated better memory retention.

### 2.8 Active Shuttle Avoidance Test (AAT)

In this experiment, a 35 (W) × 17 × 20 (H) cm^3^ aluminum box (shuttle cage, Coulbourn Instruments Model E10‐15) was used, divided into two chambers separated by a small guillotine door (7.5 (W) × 6.5 cm^2^) that allowed passage between them. The floor of the box had metal rods arranged in parallel at 1 cm intervals, connected to a current generator. The entire experimental process, including timing, light and sound stimuli, and electric shocks, was controlled using a computer program. During the test, a mouse was placed in one chamber and allowed to acclimate for 10 s. Then, a 10‐s light and sound stimulus (conditioned stimulus; CS) was presented. If the mouse stayed in the same chamber without reacting, a 5‐s electric shock of 0.3 mA (unconditioned stimulus; UCS) was administered. If the mouse moved to the other chamber during CS, no shock was given. Each mouse received five CS and UCS tests and was then returned to its cage. After a 15–20 min interval, the same procedure was repeated four times, continuing for three consecutive days. This test assessed the number of avoidance responses under the CS condition to determine the effect of different diets on the learning and memory abilities of mice.

### 2.9 The Biochemical Parameters Analysis

Blood samples were rapidly collected from the mice for biochemical analyses. Serum was separated by centrifugation at 3500 rpm for 15 min and immediately subjected to analysis without freezing. The concentrations of glucose, total protein, albumin, triglyceride, total cholesterol, high‐density lipoprotein (HDL), low‐density lipoprotein (LDL), aspartate transaminase (AST), alanine transaminase (ALT), blood urea nitrogen (BUN), and creatinine were determined using enzymatic colorimetric methods on an Olympus AU2700 (Beckman Coulter) automated analyzer with commercially available reagents.

### 2.10 Oxidative Stress Analysis

#### 2.10.1 8‐Hydroxy‐2′‐Deoxyguanosine (8‐OHdG) ELISA Assay

8‐OHdG levels in the brain were determined using an ELISA kit. Briefly, DNA was extracted using the Blood and Tissue Genomic DNA Extraction Miniprep System. DNA samples from brain tissue or standard (50 μL) were added to each well and mixed with 50 μL of the primary antibody solution. The plates were incubated overnight at 4°C. The liquid was poured out, and the sample was washed three times with 250 μL of the Washing Solution. Then, 100 μL of the Secondary Antibody Solution was added, and the mixture was incubated at room temperature for 1 h. Subsequently, the liquid was poured out again and washed three times with 250 μL of Washing Solution. In a dark environment, 100 μL of reconstitution enzyme substrate was added and left for 15 min, during which the color changed from dark blue to light blue. Finally, 100 μL of reaction‐terminating solution was added, causing the color to change to bright yellow in a dark environment. The absorbance was measured at a wavelength of 450 nm.

#### 2.10.2 Thiobarbituric Acid‐Reactive Substances (TBARS) Assay

Malondialdehyde (MDA) is a secondary product of lipid peroxidation, which reacts with 2‐thiobarbituric acid (TBA), forming a complex that can be observed spectrophotometrically at 535 nm. Brain tissue from the mice was placed on ice, and 20 times the volume of 50 mM phosphate buffer (pH 7.0) was added for dilution. The mixture was homogenized at 1400 rpm using a homogenizer to prepare the brain tissue solution. Brain tissue solution (150 μL) or MDA standards at concentrations of 0.625, 1.25, 2.5, 5, 10, and 20 μM were mixed with 300 μL TBA coloring agent and 45 μL butylated hydroxytoluene (BHT). After shaking for 1 min, the mixture was incubated in a water bath at 90°C for 45 min. The samples or standards were removed and cooled to room temperature, followed by the addition of an equal volume of n‐butanol for extraction. After shaking for 1 min, the mixture was centrifuged at 3000 rpm at 4°C for 5 min. The absorbance of the upper layer, containing the MDA derivative or MDA standard, was then measured at 535 nm using a spectrophotometer (Beckman DU530). Sample concentrations were calculated using a standard curve of known standards.

#### 2.10.3 Protein Carbonyl Content Assay

The brain tissue was placed in a plastic dish, and a homogeneous buffer solution was added. The brain tissue was cut into thin slices and allowed to stand at room temperature for 15 min to separate the protein‐containing solution. Centrifuge at 6000 × g to remove impurities. Determine the nucleic acid content on the ratio of absorbance at 280/260 nm. If the concentration was too high, 10% streptomycin sulfate was added until the final concentration was 1%. The tubes were allowed to stand at room temperature for 10 min and then centrifuged at 6000 × g. The supernatant was separated from the precipitate and dissolved. The mixture was vortexed and allowed to stand at room temperature for 10 min. The samples were scanned at wavelengths of 335–390 nm (with a blank tube as the blank), and the carbonyl content was obtained from the maximum absorbance peak.

### 2.11 Antioxidant Enzymes Activity

#### 2.11.1 Preparation of Liver Homogenate

After the mice were sacrificed, liver tissues were collected. A 0.15 g portion of the liver was excised and placed into 250 μL of 50 mM sodium phosphate buffer (pH 7.4). The sample was then homogenized for 30 s at 1400 rpm using a Polytron PT 3000 homogenizer on ice to obtain the initial liver homogenate. For final preparation, 40 μL of the homogenate was mixed with 56 μL of 50 mM sodium phosphate buffer (pH 7.4) and 96 μL of 2% Triton X‐100. The mixture was thoroughly vortexed and centrifuged at 4°C for 5 min using a high‐speed refrigerated centrifuge (Hermle Z383K). The resulting supernatant was collected and used as the final liver homogenate for subsequent analyses.

#### 2.11.2 Superoxide Dismutase (SOD) Assay

SOD activity was determined using an SOD assay kit (RANDOX, Cat. NO. SD125, UK). Aliquot 0.1 mL of liver homogenate and add 400 μL of chilled deionized water and mix thoroughly. The mixture was stored at 4°C for 15 min and then centrifuged again. Subsequently, 10 μL of the supernatant was pipetted, and 490 μL of sample diluent was added to ensure thorough mixing. The absorbance value of the sample was measured at a wavelength of 343 nm.

#### 2.11.3 Catalase (CAT) Assay

The liver homogenate was centrifuged at 10,000 × g for 5 min at 4°C. The supernatant (10 μL) was diluted 1000‐fold with 50 mM sodium phosphate buffer (pH 7.0). Next, 2 mL of the diluted organ homogenate was mixed with 1 mL of 30 mM H_2_O_2_. Absorbance was immediately measured at 240 nm and 25°C using a spectrophotometer (Beckman DU530). Absorbance was recorded every 15 s for 1 min to determine the change in absorbance. One unit of catalase was defined as the amount of enzyme that decomposes one micromole of H_2_O_2_ per min.

#### 2.11.4 Glutathione Peroxidase (GPx) Assay

Liver homogenates were prepared, followed by the preparation of a coupling reagent. The hydroperoxide substrate was subsequently prepared using Tris–HCl buffer. For the assay, 10–100 μL of the sample preparation was mixed with 875–965 μL of the coupling reagent. After thorough mixing, the hydroperoxide substrate was added to initiate the reaction. The decrease in absorbance was measured over 1–2 min using a spectrophotometer.

### 2.12 Histopathological Assessment of Brain Tissue

The extent of Aβ plaque deposition in brain tissue was evaluated by measuring the percentage of Aβ‐positive area. The assessment focused on comparing the percentage of Aβ deposition area and the number of Aβ plaques between the control group and various treatment groups in SAMP8 mice. A computer‐assisted image analysis system (Leica Q500, Germany) was used to quantify Aβ deposition under a microscope at 20× magnification. The percentage of Aβ accumulation was calculated using the following formula:

$$\left(\mathrm{A\beta}-\text{positive area}/\text{total brain area}\right)\times 100\%$$

### 2.13 Statistical Analysis

The data obtained in this study were statistically analyzed using SPSS software, and the results are expressed as the mean ± standard error of the mean (mean ± SEM). Differences among multiple groups were assessed using one‐way ANOVA variance, followed by Duncan's Multiple Range Test for group comparisons. A *p*‐value of less than 0.05 indicated statistical significance. For survival analysis, Kaplan–Meier survival curves were generated, and differences between groups were evaluated using the Log‐rank test, with a *p*‐value of less than 0.05 considered statistically significant.

## 3 Results

### 3.1 Effect of Algal‐Derived Omega‐3 DHA on the SAMP8 Survival Test

**FIGURE 2:**
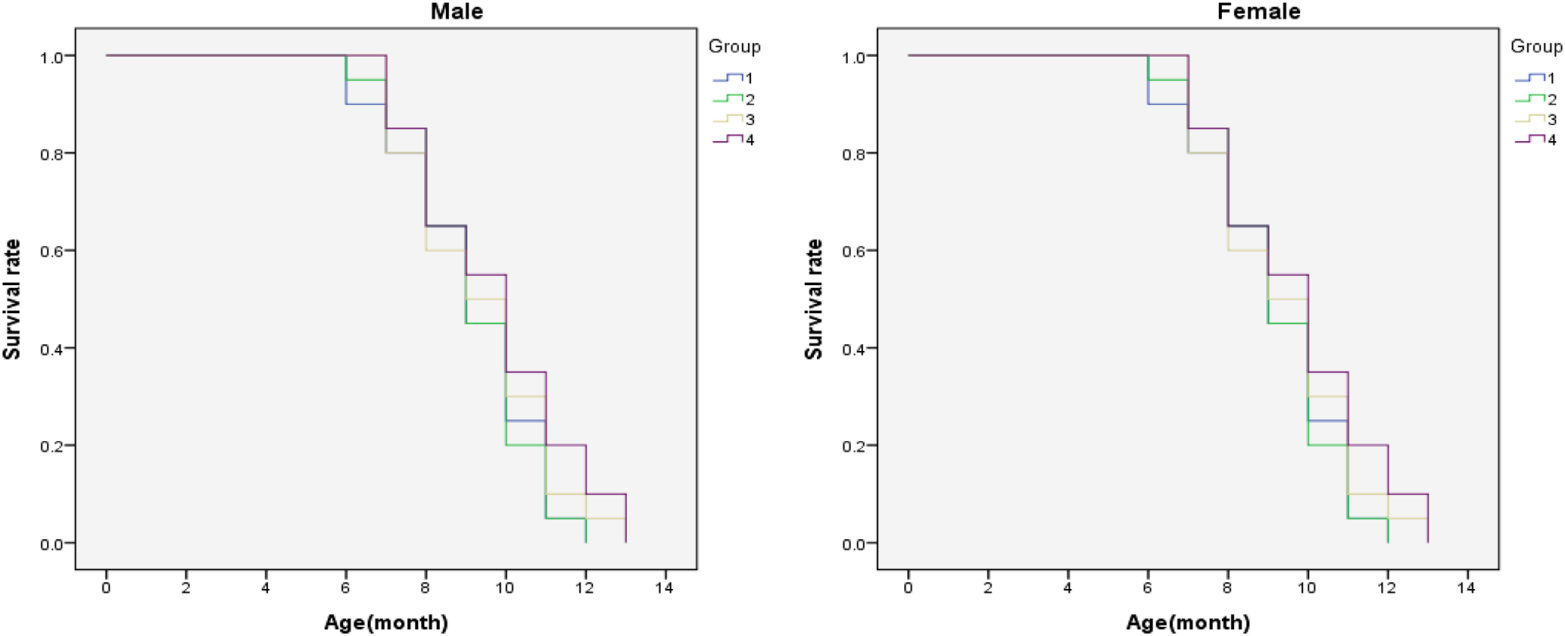
The survival rate of 6‐month‐old SAMP8 mice during algal omega3‐DHA treatment. 1: Control; 2: Algal Omega3‐DHA low dosage treatment (123 mg/kg BW/day); 3: Algal Omega3‐DHA medium treatment (246 mg/kg BW/day); 4: Algal Omega3‐DHA high dosage treatment (492 mg/kg BW/day).

Survival rates of 6‐month‐old SAMP8 mice in the control group and each experimental group (Figure 2). The results indicated that in the control group, the median age of death for 50% of the male mice was 9.0 months and that for female mice was 9.1 months. In the algal‐derived Omega‐3 DHA‐treated group, the average lifespans for males were 9.0 months in the low‐dose group, 9.2 months in the medium‐dose group, 9.3 months, and 9.5 months, respectively. The average lifespans for females were 9.1 months, 9.4, and 9.7 months, respectively.

### 3.2 Body Weight, Food Intake, Water Intake and Locomotion Activity in SAMP8 Mice

Body weight and average food intake of male and female SAMP8 mice after 13 weeks of feeding with the test substance (Table 1). Across all the groups, there were no significant differences in body weight, food intake, or water intake before and after the experiment. Specifically, algal‐derived Omega‐3 DHA supplementation did not significantly affect these parameters (*p* > 0.05), and body weight remained stable throughout the experimental period. As shown in Table 2, there were no significant differences in locomotor activity among groups during both the 0–5 min and 6–10 min intervals. A general decline in activity over time was observed, which may be attributed to the mice's initial exploratory behavior in response to the novel environment. In the first few minutes, mice typically exhibit increased horizontal movement due to heightened exploration. As they gradually adapt to the surroundings after approximately 5 min, their activity levels tend to decrease. As reported in our earlier work, Astragalus extract and its complex exhibited no significant effects on these parameters after 8 weeks, with body weight, food intake, and water consumption remaining stable (Chou et al. 2023). Similarly, supplementation with rAPS at varying doses (1×, 2×, and 5× the recommended dose) showed no significant changes in body weight or dietary intake over a 42‐day trial (Wong et al. 2024). However, a slight but nonsignificant decrease in body weight was observed in SAMP8 mice treated with the chlorella and lion's mane mushroom complex, with food and water intake remaining stable (Chou et al. 2022). Collectively, these results indicate the metabolic stability and physiological safety of these supplements. Notably, the minor weight reduction observed in the chlorella and lion's mane groups suggests potential metabolic regulatory effects, distinguishing it from other interventions.

**TABLE 1:** The changes of body weight, food intake, and water consumption in 6‐month‐old SAMP8 mice after 13 weeks of algal omega3‐DHA treatment.^1^

Groups	Body weight (g)			Food intake (g/day)	Water consumption (mL/day)
	Initial	Final	Gain		
Male					
A	29.19^a^ ± 0.24	30.33^a^ ± 0.28	1.13^a^ ± 0.10	4.66^a^ ± 0.06	6.33^a^ ± 0.09
B	28.74^a^ ± 0.21	29.98^a^ ± 0.22	1.24^a^ ± 0.08	4.58^a^ ± 0.08	6.29^a^ ± 0.07
C	28.93^a^ ± 0.23	29.94^a^ ± 0.20	1.02^a^ ± 0.14	4.63^a^ ± 0.05	6.25^b^ ± 0.06
D	29.02^a^ ± 0.29	30.13^a^ ± 0.27	1.11^a^ ± 0.08	4.52^a^ ± 0.07	6.21^c^ ± 0.07
E	29.21^a^ ± 0.21	30.41^a^ ± 0.20	1.20^a^ ± 0.11	4.60^a^ ± 0.05	6.17^d^ ± 0.07
Female					
A	26.10^a^ ± 0.43	27.20^a^ ± 0.39	1.10^a^ ± 0.12	4.34^a^ ± 0.05	6.20^a^ ± 0.09
B	25.92^a^ ± 0.59	26.98^a^ ± 0.63	1.06^a^ ± 0.14	4.27^a^ ± 0.06	6.02^b^ ± 0.12
C	26.19^a^ ± 0.44	27.13^a^ ± 0.44	0.94^a^ ± 0.15	4.30^a^ ± 0.06	6.03^b^ ± 0.06
D	26.13^a^ ± 0.39	27.25^a^ ± 0.42	1.12^a^ ± 0.08	4.36^a^ ± 0.05	5.91^b^ ± 0.08
E	25.86^a^ ± 0.36	26.84^a^ ± 0.35	0.98^a^ ± 0.10	4.37^a^ ± 0.06	6.02^b^ ± 0.09

*Note:* A: Control; B: Algal Omega3‐DHA 1× treatment (246 mg/kg BW/day); C: Algal Omega3‐DHA 2× treatment (492 mg/kg BW/day); D: Algal Omega3‐DHA + PC 1× treatment (246 mg/kg BW/day); E: Algal Omega3‐DHA + PS 1× treatment (246 mg/kg BW/day).

^1^ Values were expressed as mean ± SEM and analyzed by one‐way ANOVA.

**TABLE 2:** The change of locomotion in 6‐month‐old SAMP8 mice after 10 weeks of algal omega3‐DHA treatment.^1^ ^,^ ^2^

Groups	Locomotion (time interval [mins])	
	0–5	6–10
Male	(sec/5 min)	
A	92.42^a^ ± 1.76	81.67^a^ ± 1.81
B	91.92^a^ ± 1.64	81.08^a^ ± 1.88
C	93.17^a^ ± 1.78	80.33^a^ ± 1.72
D	92.75^a^ ± 1.62	80.75^a^ ± 1.86
E	93.33^a^ ± 1.59	81.92^a^ ± 1.64
Female		
A	82.33^a^ ± 1.59	71.67^a^ ± 1.64
B	80.33^a^ ± 1.81	72.17^a^ ± 1.42
C	80.50^a^ ± 1.75	70.92^a^ ± 1.89
D	81.75^a^ ± 1.74	71.42^a^ ± 1.89
E	82.08^a^ ± 1.88	71.50^a^ ± 1.82

*Note:* A: Control. B: Algal Omega3‐DHA 1× treatment (246 mg/kg BW/day). C: Algal Omega3‐DHA 2× treatment (492 mg/kg BW/day). D: Algal Omega3‐DHA + PC 1× treatment (246 mg/kg BW/day). E: Algal Omega3‐DHA + PS 1× treatment (246 mg/kg BW/day).

^1^ Values were expressed as mean ± SEM and analyzed by one‐way ANOVA.

^2^ Record time indicated every 5‐min reading by the monitor E61‐21.

### 3.3 Effect of Algal‐Derived Omega3‐DHA on Organ Weight

After 13 weeks of feeding male and female SAMP8 mice the test substances, the relative weights of the brain, heart, liver, spleen, lungs, and kidneys showed no significant differences between the experimental and control groups (Table S1). Visual examination upon sacrifice revealed no abnormalities such as organ enlargement, hard lumps, or discoloration. Specifically, algal‐derived Omega‐3 DHA supplementation did not significantly affect organ weights, including the liver (1.25 ± 0.05 g) and kidneys (0.28 ± 0.02 g) (*p* > 0.05), demonstrating its metabolic stability and physiological safety. Similarly, Astragalus extract and its complex showed no significant differences in liver and kidney weights (Chou et al. 2023). Supplementation with rAPS also yielded consistent results (Wong et al. 2024). Similarly, the chlorella and lion's mane mushroom complex did not significantly alter liver or kidney weights (Chou et al. 2022). Collectively, these findings confirmed the metabolic stability and physiological safety of these supplements. While none of the interventions significantly affected organ weights, the additional neuroprotective effects observed with the chlorella and lion's mane complex, as well as the anti‐fatigue properties of rAPS, highlight the diverse functional benefits of these dietary supplements.

### 3.4 Effect of Algal Omega3‐DHA on the Aging Index

The aging index was used to evaluate the degree of aging in SAMP8 mice, with assessment items including behavioral and physical appearance changes. Each item is scored on a scale of 0 to 4, with higher scores indicating more severe aging. After 11 weeks of feeding male and female SAMP8 mice with the test substance (Table S2), the results showed that for male mice, the total aging index scores of all experimental groups were significantly lower than those of the control group (*p* < 0.05). In female mice, the total aging index scores of groups C, D, and E were significantly lower than those of the control group (*p* < 0.05). Similarly, Astragalus extract and its complex have been reported to significantly reduce total aging scores of SAMP8 mice, further supporting their antiaging potential (Chou et al. 2023).

In this study, algal‐derived Omega‐3 DHA significantly reduced the aging index in SAMP8 mice, particularly when combined with PC and PS. This finding aligns with that of Reale et al. (2022), who demonstrated that alterations in the cholinergic system and increased inflammation are associated with aging in SAMP8 mice. Our study further demonstrated that algal‐based DHA led to significant improvements in both behavioral performance and physiological aging indices. Zhao et al. (2020) found that DHA combined with phospholipids effectively restores lipid metabolism in aging mice, further reducing aging‐related indices. This supports our findings on the synergistic effects of DHA and phospholipids in lowering the aging index of SAMP8 mice.

### 3.5 Effect of Algal Omega3‐DHA on Hematological Parameters

After 13 weeks of algal omega‐3 DHA supplementation, hematological parameters were measured in 6‐month‐old SAMP8 mice (Table S3). In male mice, there were no significant differences in glucose, total protein, albumin, triglyceride, total cholesterol, high‐density lipoprotein (HDL), low‐density lipoprotein (LDL), aspartate transaminase (AST), alanine transaminase (ALT), blood urea nitrogen (BUN), or creatinine levels across the groups (*p* > 0.05). However, slight trends were observed. Group C (algal DHA 2×) showed the lowest mean glucose level (112.67 ± 3.72 mg/dL), while Group E (algal DHA + PS) exhibited the highest HDL level (57.67 ± 3.24 mg/dL) and lowest LDL (7.15 ± 0.27 mg/dL), suggesting a modest improvement in lipid profiles. In female mice, similar trends were observed. Group E showed the lowest LDL (7.13 ± 0.17 mg/dL) and relatively higher HDL (54.42 ± 2.87 mg/dL) compared to the control. Liver function markers (AST and ALT) remained stable across all groups in both sexes, with no signs of hepatotoxicity. Likewise, renal function indicators, including BUN and creatinine, showed no abnormalities, indicating the safety of long‐term algal DHA supplementation.

### 3.6 Effect of Algal‐Derived Omega3‐DHA on Memory Retention and Learning Enhancement

**FIGURE 3:**
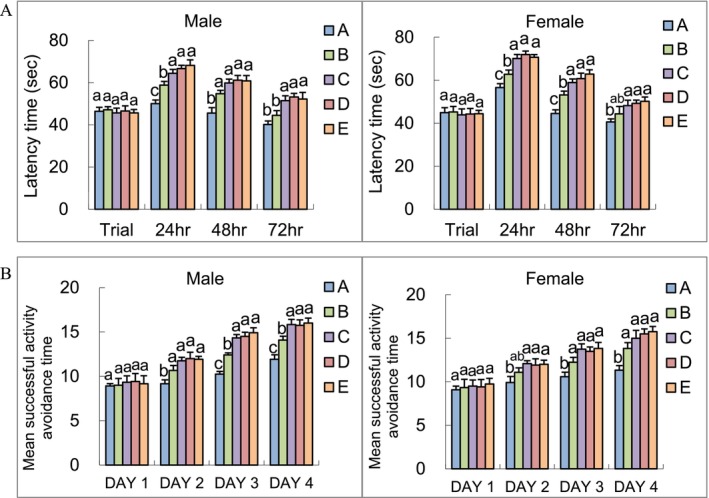
Single‐trial passive avoidance ability (A) and active avoidance ability (B) of 6‐month‐old SAMP8 mice after 12 weeks (for PAT) and 13 weeks (for AAT) of algal omega3‐DHA treatment^1,2^. ^1^Values were expressed as mean ± SEM and analyzed by one‐way ANOVA (*n* = 12). ^2^Groups with different letters indicate significant differences among each group (*p* < 0.05). A: Control. B: Algal Omega3‐DHA 1× treatment (246 mg/kg BW/day). C: Algal Omega3‐DHA 2× treatment (492 mg/kg BW/day). D: Algal Omega3‐DHA + PC 1× treatment (246 mg/kg BW/day). E: Algal Omega3‐DHA + PS 1× treatment (246 mg/kg BW/day).

After 13 weeks of feeding male and female SAMP8 mice the test substances, body weight and food intake measurements showed no significant differences across groups, indicating metabolic stability during the experimental period (Table S1). To evaluate the cognitive performance of the mice, both the single‐trial Passive Avoidance Test (PAT) (Figure 3A) and the Active Avoidance Test (Figure 3B) were conducted. In the PAT, the retention time in the light chamber was measured, with longer times reflecting better memory retention.

After 12 weeks of feeding male and female SAMP8 mice with the test substance, their learning and memory abilities were assessed using PAT, which measures retention time in the light chamber. Longer retention times indicated better learning and memory. The results showed that for male mice, at 24 h, all experimental groups had significantly longer retention times than the control group, with groups C, D, and E showing the longest retention times (*p* < 0.05). At 48 h, the experimental groups had significantly longer retention times than the control group (*p* < 0.05). At 72 h, groups C, D, and E were significantly longer compared to the control group (*p* < 0.05). At 24 h, all experimental groups had significantly longer retention times than the control group (*p* < 0.05). At 48 h, the experimental groups showed significantly longer retention times, with groups C, D, and E having the longest retention times (*p* < 0.05). At 72 h, groups C, D, and E had significantly longer retention times compared to the control group (*p* < 0.05).

After 13 weeks of feeding male and female SAMP8 mice the test substance, their learning and memory abilities were assessed using the active avoidance test, where a higher number of successful avoidance responses indicated better cognitive function. For male mice, there was no significant difference between the groups on the first day as the mice were still in the learning phase. However, on the second day, all experimental groups showed significantly more successful avoidance responses than the control group (*p* < 0.05). On the third day, the experimental groups showed a significant increase in successful avoidance responses compared with the control group, with groups C, D, and E having the highest numbers (*p* < 0.05). By the fourth day, all experimental groups had significantly more successful avoidance responses than did the control group (*p* < 0.05). In female mice, there was no significant difference between the groups on the first day. On the second day, groups C, D, and E showed significantly more successful avoidance responses than the control group (*p* < 0.05). On the third day, all experimental groups had significantly more successful avoidance responses than the control group (*p* < 0.05). By the fourth day, all experimental groups had significantly more successful avoidance responses than did the control group (*p* < 0.05).

### 3.7 Effect of Algal Omega3‐DHA on Brain Oxidative Stress Reduction

**FIGURE 4:**
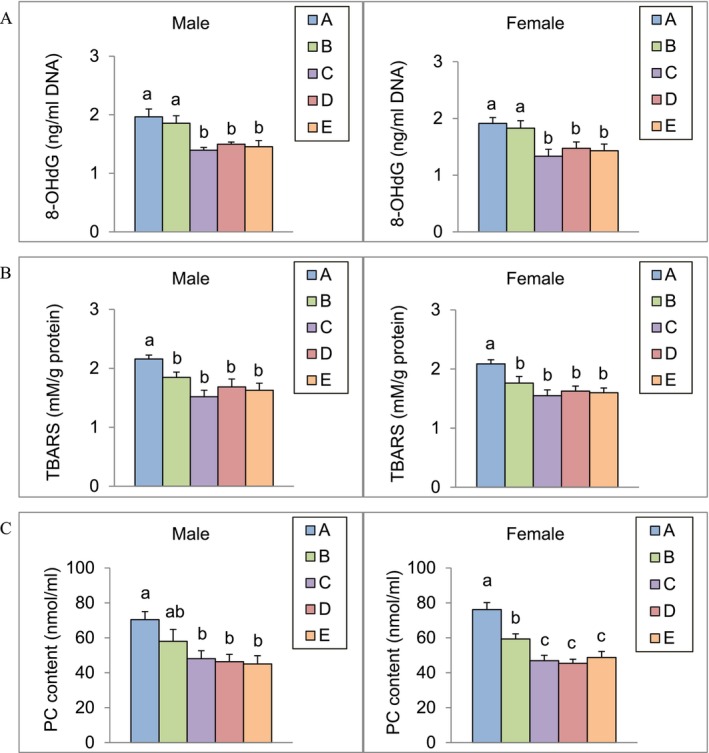
The 8‐OHdG (A), TBARS (B), and protein carbonyl content (C) in SAMP8 mice brains after 13 weeks of algal omega3‐DHA treatment^1,2^. ^1^Values were expressed as mean ± SEM and analyzed by one‐way ANOVA (*n* = 12). ^2^Groups with different letters indicate significant differences among each group (*p* < 0.05). A: Control. B: Algal Omega3‐DHA 1× treatment (246 mg/kg BW/day). C: Algal Omega3‐DHA 2× treatment (492 mg/kg BW/day). D: Algal Omega3‐DHA + PC 1× treatment (246 mg/kg BW/day). E: Algal Omega3‐DHA + PS 1× treatment (246 mg/kg BW/day).

After 13 weeks of feeding male and female SAMP8 mice with the test substance, brain levels of 8‐OHdG, lipid peroxides, and protein carbonyl content were analyzed (Figure 4). The results showed that the levels of 8‐OHdG in the brains of both male and female mice in groups C, D, and E were significantly lower than those in the control group (*p* < 0.05). TBARS levels in the brains of all experimental groups were significantly lower than those in the control group (*p* < 0.05). Additionally, the brain protein carbonyl content in male mice in groups C, D, and E was significantly lower than that in the control group (*p* < 0.05). In female mice, the brain protein carbonyl content in all experimental groups was lower than that in the control group, with groups C, D, and E showing the most significant reduction (*p* < 0.05).

The reduction in markers, such as 8‐OHdG, TBARS, and protein carbonyl content, highlights the efficacy of DHA in combating oxidative damage. These results align with the findings of Díaz et al. (2021), who emphasized the role of DHA as an indirect antioxidant that enhances the oxidative defense system of the brain by regulating gene expression. Faccinetto‐Beltrán et al. (2024) demonstrated that DHA supplementation combined with other neuroprotective agents reduces oxidative stress markers in a rat model of cognitive impairment. Their findings support our conclusion that DHA, especially when combined with phospholipids, such as PC or PS, offers synergistic effects in reducing oxidative stress in the brain. Furthermore, Lauritzen et al. (2016) highlighted the ability of DHA to mitigate lipid peroxidation and protect neuronal membranes, thereby preserving brain function during aging. Our study corroborates these findings as algal‐based DHA reduced TBARS levels, a critical indicator of lipid peroxidation. Zhao et al. (2020) found that DHA‐enriched phospholipids restored essential lipid profiles in SAMP8 mice, particularly by improving the levels of DHA‐containing plasmalogens, which are known to be critical in reducing oxidative stress. Our results extend this understanding by showing that algal‐derived DHA with phospholipids further amplifies the antioxidative effects.

### 3.8 Effect of Algal Omega3‐DHA on Liver Antioxidant Enzyme Activity

**FIGURE 5:**
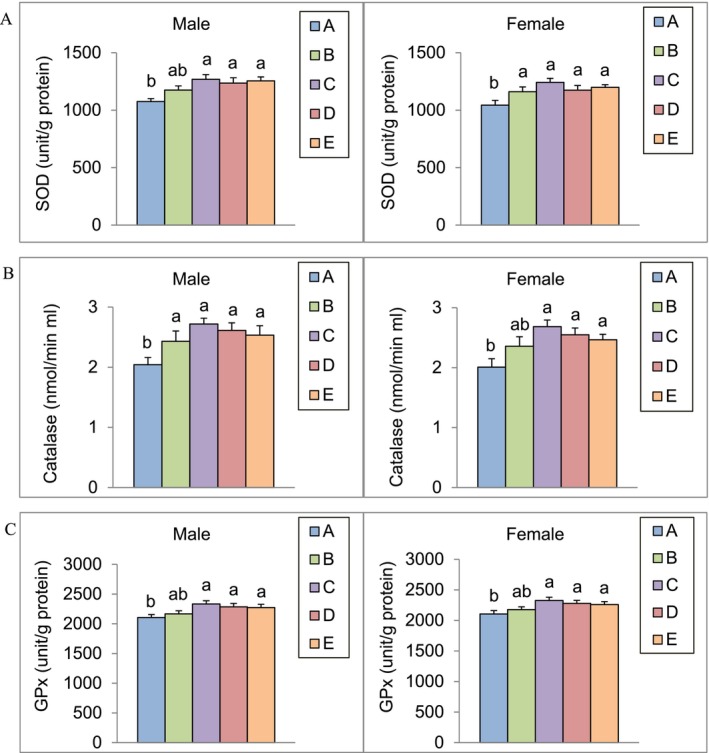
SOD activity (A), CAT activity (B) and GPx activity (C) in SAMP8 mice livers after 13 weeks of algal omega3‐DHA treatment^1,2^. ^1^Values were expressed as mean ± SEM and analyzed by one‐way ANOVA (*n* = 12). ^2^Groups with different letters indicate significant differences among each group (*p* < 0.05). A: Control. B: Algal Omega3‐DHA 1× treatment (246 mg/kg BW/day). C: Algal Omega3‐DHA 2× treatment (492 mg/kg BW/day). D: Algal Omega3‐DHA + PC 1× treatment (246 mg/kg BW/day). E: Algal Omega3‐DHA + PS 1× treatment (246 mg/kg BW/day).

After 13 weeks of feeding male and female SAMP8 mice with the test substance, the liver activities of SOD, CAT, and GPx were analyzed (Figure 5). The results showed that SOD activity in male mice from groups C, D, and E was significantly higher than that in the control group (*p* < 0.05). Similarly, SOD activity in female mice was significantly higher in all experimental groups than that in the control group (*p* < 0.05). Catalase activity was significantly higher in male mice in all experimental groups than that in the control group (*p* < 0.05). In female mice, the catalase activity in groups C, D, and E was significantly higher than that in the control group (*p* < 0.05). Finally, GPx activity in the liver was significantly higher in both male and female mice in groups C, D, and E than in the control group (*p* < 0.05).

### 3.9 Effect of Algal Omega3‐DHA on Aβ Participation in SAMP8 Mice Brains

**FIGURE 6:**
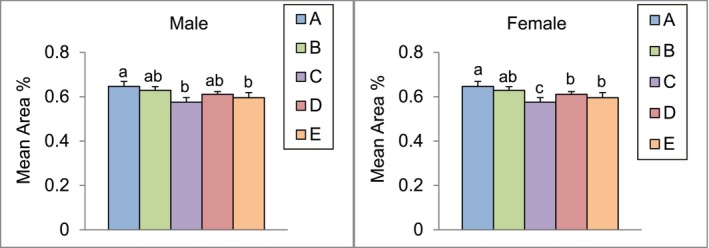
The Aβ participation area (%) in 6‐month‐old SAMP8 mice brains after 13 weeks of algal omega3‐DHA treatment^1^. ^1^Values were expressed as mean ± SEM and analyzed by one‐way ANOVA (*n* = 12). A: Control. B: Algal Omega3‐DHA 1× treatment (246 mg/kg BW/day). C: Algal Omega3‐DHA 2× treatment (492 mg/kg BW/day). D: Algal Omega3‐DHA + PC 1× treatment (246 mg/kg BW/day). E: Algal Omega3‐DHA + PS 1× treatment (246 mg/kg BW/day).

After 13 weeks of feeding male and female SAMP8 mice with the test substance, the percentage of the brain area occupied by Aβ protein deposits was analyzed (Figure 6). The results showed that the percentage of brain area with Aβ protein deposits was reduced in both male and female mice in the experimental groups compared to that in the control group. Among all treatments, the Algal Omega‐3 DHA 2× group exhibited the greatest reduction in Aβ deposition.

## 4 Discussion

### 4.1 Enhanced Bioavailability and Synergistic Effects of DHA With Phospholipids (PC and PS)

Our study provides strong evidence that algal‐derived Omega‐3 DHA, particularly when combined with PC and PS, significantly enhances cognitive function, neuroprotection, and antioxidant enzyme activity in aging SAMP8 mice. Results from passive and active avoidance tests demonstrated that DHA supplementation markedly improved memory retention and learning ability (*p* < 0.05), reinforcing its role in cognitive preservation. The DHA‐PL formulation demonstrates superior absorption and integration into neuronal membrane compared to conventional DHA‐TAG from fish oil (Zhao et al. 2020), and this enhanced bioavailability and transport efficiency across the BBB makes DHA‐PL a more effective strategy for neurological applications. Compared to DHA‐TAG, DHA‐PL exhibited a greater ability to mitigate age‐related cognitive decline, as evidenced by reductions in neurodegenerative markers, including Aβ accumulation and oxidative stress indicators. These findings align with previous research demonstrating DHA's role in synaptic plasticity, neurogenesis, and brain‐derived neurotrophic factor (BDNF) regulation, further validating its neuroprotective potential (Cole and Frautschy 2010). Our results further indicate that DHA‐PS and DHA‐PC supplementation significantly improved learning and memory retention in SAMP8 mice, as evidenced by their superior performance in passive and active avoidance tests (*p* < 0.05). Additionally, these formulations significantly reduced oxidative stress markers (8‐OHdG, TBARS, and protein carbonyl content) while enhancing antioxidant enzyme activity (SOD, CAT, GPx), suggesting a potent neuroprotective effect. The ability of DHA‐PS and DHA‐PC to enhance DHA uptake and retention within neuronal membranes suggests their potential superiority over conventional DHA supplements, enabling a more targeted and sustained release of DHA in the brain.

These findings support the application of DHA‐PS and DHA‐PC in functional foods, dietary supplements, and precision medicine approaches for neurodegenerative diseases, positioning them as next‐generation solutions for brain health and healthy aging. By improving cognitive function, reducing oxidative damage, and enhancing neuroprotection, phospholipid‐bound DHA formulations present a promising strategy for preventing age‐related neurological decline and optimizing long‐term brain health.

### 4.2 Algal‐Based DHA in Reducing Oxidative Stress and Inflammation

Our study confirms that algal‐based DHA exhibits potent antioxidant properties, significantly reducing oxidative stress markers, including 8‐OHdG, TBARS, and protein carbonyl content (*p* < 0.05). These findings indicate that algal‐based DHA effectively neutralizes oxidative damage, a key contributor to neurodegeneration and aging‐related diseases such as Alzheimer's and Parkinson's disease. Oxidative stress accelerates cellular aging by inducing mitochondrial dysfunction, lipid peroxidation, and chronic neuroinflammation, all of which contribute to cognitive decline and neurodegeneration. By significantly reducing oxidative markers, algal‐based DHA may slow disease progression and enhance neuronal resilience, making it a promising therapeutic approach for age‐related neurological disorders.

A major advantage of plant‐based DHA over fish‐derived DHA is its unique phytochemical profile, which includes naturally occurring polyphenols, flavonoids, and carotenoids. These bioactive compounds work synergistically with DHA to enhance oxidative stress protection by acting as free radical scavengers and inflammation modulators. Moreover, algal‐based DHA significantly increases antioxidant enzyme activity, including SOD, CAT, and GPx, reinforcing its systemic protective effect against oxidative stress. Mitochondrial dysfunction, a major hallmark of neurodegeneration, leads to adenosine triphosphate (ATP) depletion and excessive reactive oxygen species (ROS) production. By maintaining mitochondrial efficiency, enhancing antioxidant defenses, and reducing oxidative burden, algal DHA contributes to neuronal longevity and supports overall brain energy metabolism.

Islam et al. (2020) reported that DHA supplementation improved the oxidative profile of dementia models by increasing antioxidant enzyme activity. Another study highlighted the role of DHA as a master regulator of cellular antioxidant defenses, promoting the transcriptional regulation of antioxidant enzymes through nuclear factor erythroid 2‐related factor 2 (Nrf2) activation (Borgonovi et al. 2023).

In this study, we observed that algal‐based Omega‐3 DHA significantly reduced Aβ accumulation in the brains of SAMP8 mice, consistent with findings from other recent studies. For instance, the study demonstrated that DHA supplementation decreased Aβ levels and improved cognitive function in aged mice by modulating lipid metabolism and enhancing synaptic plasticity (Zhang, Yuan, et al. 2024; Zhang, Huang, and Shi 2024). Furthermore, the neuroprotective effects of DHA have been reported to be mediated through anti‐inflammatory pathways. Lista et al. (2024) found that DHA supplementation attenuated neuroinflammation and Aβ pathology in a mouse model of AD by modulating microglial activity. These studies support our findings, indicating that algal‐based DHA effectively reduces Aβ accumulation in SAMP8 mice. Oxidative stress plays a central role in cellular aging, leading to DNA damage, mitochondrial dysfunction, and chronic inflammation, all of which accelerate age‐related cognitive and physiological decline (Stojanovic et al. 2025). In addition to its antioxidative properties, algal‐derived DHA supplementation was found to significantly reduce Aβ accumulation and neuroinflammatory responses, indicating its potential role in preventing neurodegenerative disorders such as AD (Zhang, Yuan, et al. 2024; Zhang, Huang, and Shi 2024).

These studies support our results, indicating that algal‐based DHA can effectively enhance liver antioxidant defense. Future studies should further explore clinical applications in human populations, particularly in individuals at high risk for cognitive decline and neurodegenerative diseases, to validate its therapeutic potential.

### 4.3 Longevity and Survival Extension in Aging SAMP8 Mice

Our study demonstrated that algal‐based DHA supplementation significantly extended the lifespan of SAMP8 mice. The survival test results revealed a notable increase in median survival among DHA‐treated groups, particularly in high‐dose male and female mice (*p* < 0.05). Additionally, the aging index, which assesses behavioral and physiological aging markers, was significantly reduced across all experimental groups, suggesting a delay in age‐related functional decline. The lifespan extension effect of algal‐based DHA may be attributed to its ability to reduce systemic oxidative stress, neuroinflammation, and metabolic dysregulation—three major contributors to aging and neurodegeneration. Aβ plaques are a hallmark of Alzheimer's pathology, contributing to synaptic dysfunction, neurotoxicity, and cognitive decline. The reduction in Aβ deposition observed in algal‐based DHA‐treated groups suggests that algal‐based DHA may enhance neuronal resilience and promote synaptic integrity.

Kuratko et al. (2013) highlighted DHA as a key lipid in the brain essential for learning and behavior, while Lauritzen et al. (2016) emphasized its importance for neuronal growth, differentiation, and synaptic signaling across the lifespan. Additionally, Islam et al. (2020) reported that DHA supplementation restored brain lipid composition and improved cognition in dementia models, supporting our findings that algal‐derived DHA, particularly when combined with PC or PS, enhances cognitive abilities. Taoro‐González et al. (2022) demonstrated the capacity of DHA to improve behavioral performance in aging models, consistent with our results. Comparable results were observed in our earlier work with Astragalus extract and its complexes, which increased retention time in the light chamber during the PAT, demonstrating its neuroprotective and cognitive‐enhancing potential (Chou et al. 2023).

Algal‐based DHA emerges as a promising, sustainable alternative for lifespan extension and cognitive health preservation. Given the global shift toward plant‐based nutraceuticals and sustainable healthcare solutions, algal‐based DHA represents a viable, eco‐friendly substitute for conventional fish oil‐derived DHA, with broader implications for age‐related disease prevention and personalized nutrition strategies. Further clinical research is warranted to explore its translational applications in human aging and neurodegenerative disease management.

### 4.4 Comparative Advantages of Algal‐Derived DHA: Bioavailability, Safety, and Functional Potential

Although the molecular structure of DHA is identical regardless of its origin, the bioavailability and physiological impact of DHA can differ markedly depending on its lipid‐bound form and source. Marine‐derived DHA is commonly present in TAG or EE forms, which require enzymatic hydrolysis and reesterification prior to absorption, often resulting in limited efficiency (Yurko‐Mauro et al. 2015). In contrast, the algal‐derived DHA used in this study was administered in plant‐based formulations, some of which were conjugated with PC or PS, allowing for enhanced intestinal absorption and more efficient incorporation into cellular and neuronal membranes. Previous studies have demonstrated that DHA‐PL crosses the BBB more effectively than conventional DHA, resulting in higher accumulation in brain tissue and improved neuroprotective outcomes (Zhao et al. 2020; Hachem et al. 2020).

In our SAMP8 mouse model, groups supplemented with algal DHA + PC or DHA + PS exhibited favorable trends in lipid metabolism (elevated HDL, reduced LDL) and maintained normal liver and kidney function biomarkers, further supporting the systemic safety of this form. Additionally, algal‐derived DHA offers unique advantages over marine sources by being free from environmental contaminants such as heavy metals, polychlorinated biphenyls, and microplastics (Minihane et al. 2015). It also contains plant‐based phytochemicals—such as carotenoids and polyphenols—that may enhance antioxidant and anti‐inflammatory capacity, potentially acting synergistically with DHA to reduce oxidative damage and modulate pro‐inflammatory cytokines (e.g., IL‐1β and TNF‐α) (Gutiérrez et al. 2019; Calder 2015). Taken together, these characteristics position algal‐derived DHA, particularly in phospholipid‐bound forms, as a promising, sustainable intervention for mitigating cognitive decline and supporting healthy aging. The novelty of this study lies in its focus on the efficacy of algal‐derived DHA, which not only matches but also surpasses the effects of conventional marine‐derived DHA in reducing behavioral and physical aging indices.

## 5 Conclusions

This study provides compelling evidence that algal‐derived Omega‐3 DHA supplementation exerts significant antiaging and neuroprotective effects in SAMP8 mice. By evaluating cognitive function, oxidative stress markers, antioxidant enzyme activity, and survival rates, the results demonstrated that algal Omega‐3 DHA, particularly with PC and PS, effectively mitigates age‐related cognitive decline, enhances antioxidant defenses, and extends lifespan. Cognitive function was significantly improved, as shown by the passive and active avoidance tests, where latency times and avoidance responses were markedly increased in the experimental groups. Groups C, D, and E exhibited superior memory retention compared to the controls, suggesting a neuroprotective effect in aging models. Algal Omega‐3 DHA also demonstrates potent antioxidative properties, significantly reducing oxidative stress markers in the brain. Levels of 8‐OHdG and TBARS were significantly lower in the DHA‐treated groups, indicating enhanced cellular defense against oxidative damage. Liver antioxidant enzyme activity was also significantly elevated, with increased SOD, CAT, and GPx levels compared to controls. In addition, Aβ plaque accumulation, a hallmark of neurodegeneration, was significantly reduced in the experimental groups. Notably, the median survival was significantly prolonged in the high‐dose group, underscoring the longevity‐enhancing potential of algal Omega‐3 DHA. Importantly, no adverse effects were observed on body weight, organ weight, or metabolic parameters, confirming the safety profile. In conclusion, algal‐derived Omega‐3 DHA supplementation enhances cognitive function, attenuates oxidative stress, reduces neurodegenerative markers, and extends lifespan. These findings support its potential as a sustainable algal‐based therapeutic strategy for aging‐related cognitive decline and neurodegenerative disorders, including AD.

## Author Contributions

**Ming‐Yu Chou and I‐Hung Lin:** conceptualization (equal), formal analysis (equal), funding acquisition (equal), investigation (equal). **Chia‐Jung Chen and Shih‐Yi Wang:** formal analysis (equal), funding acquisition (equal), software (equal), supervision (equal), validation (equal). **Ting‐Jian Guo and Ching‐Hsin Chi:** data curation (equal), formal analysis (equal), investigation (equal). **Che‐An Lin:** software (equal), supervision (equal), validation (equal), visualization (equal). **Dao‐Na Yang and Po‐Hsien Li:** investigation (equal), methodology (equal), validation (equal), visualization (equal). **Yu‐Chen Li:** data curation (equal), formal analysis (equal), methodology (equal), software (equal), supervision (equal). **Chieh‐Chang Tien and Ming‐Fu Wang:** data curation (equal), formal analysis (equal), resources (equal), software (equal), supervision (equal). **Ruei‐Ze Lin and Mei‐Due Yang:** formal analysis (equal), investigation (equal), validation (equal), visualization (equal).

## Ethics Statement

Animal and Human Rights Statement: All animal procedures were conducted in accordance with the standards set forth in the guidelines for the Care and Use of Experimental Animals by the Committee for the Purpose of Control and Supervision of Experiments on Animals and the National Institutes of Health. The protocol was approved by the Committee on Animal Research, Providence University, under code 20230307‐A004.

## Conflicts of Interest

The authors declare no conflicts of interest.

## Supporting information


**Tables S1‐S3** fsn371353‐sup‐0001‐TableS1‐S3.docx.

## Data Availability

The datasets used during the current study are available from the corresponding author on reasonable request.
