# Supplementary material for: Potential Anti‐Aging Effects of a Dietary Supplement From the Algal‐Derived Omega‐3 DHA in Aged SAMP 8 Mice
Source: Food Sci Nutr. 2026 Jan 30;14(2):e71353. doi: 10.1002/fsn3.71353 (PMC12859168; doi:10.1002/fsn3.71353)
Supplement: Supplementary file 1 — Tables S1‐S3 fsn371353‐sup‐0001‐TableS1‐S3.docx. [file FSN3-14-e71353-s001.docx]

**Supplementary tables**

Table S1. Comparisons of relative organs weight in 6 months-old SAMP8 mice after 13 weeks of algal omega3-DHA treatment.^1, 2^

| Groups | Relative organ weights (g/100g body weight) | | | | | |
| --- | --- | --- | --- | --- | --- | --- |
|  | Brain | Heart | Liver | Spleen | Lung | Kidney |
| **Male** |  |  |  |  |  |  |
| A | 1.46±0.03 | 0.62±0.03 | 4.78±0.12 | 0.26±0.01 | 0.66±0.02 | 1.61±0.02 |
| B | 1.48±0.02 | 0.63±0.03 | 4.97±0.11 | 0.27±0.02 | 0.69±0.02 | 1.64±0.04 |
| C | 1.52±0.02 | 0.67±0.02 | 4.95±0.09 | 0.29±0.02 | 0.67±0.02 | 1.66±0.03 |
| D | 1.50±0.03 | 0.65±0.02 | 4.88±0.10 | 0.27±0.02 | 0.64±0.02 | 1.62±0.03 |
| E | 1.45±0.03 | 0.63±0.02 | 4.80±0.13 | 0.29±0.02 | 0.68±0.02 | 1.63±0.02 |
| **Female** |  |  |  |  |  |  |
| A | 1.48±0.03 | 0.67±0.02 | 4.85±0.08 | 0.27±0.02 | 0.67±0.02 | 1.53±0.02 |
| B | 1.52±0.03 | 0.61±0.02 | 4.80±0.08 | 0.30±0.02 | 0.69±0.03 | 1.55±0.02 |
| C | 1.44±0.04 | 0.66±0.03 | 4.87±0.09 | 0.26±0.03 | 0.67±0.02 | 1.56±0.02 |
| D | 1.47±0.02 | 0.64±0.02 | 4.84±0.09 | 0.25±0.02 | 0.65±0.02 | 1.60±0.02 |
| E | 1.50±0.02 | 0.65±0.03 | 4.81±0.43 | 0.29±0.02 | 0.64±0.02 | 1.57±0.02 |

^1^ Values were expressed as mean ± SEM and analyzed by one-way ANOVA

^2^ Organ weight (%) = (weight of the organ / body weight) *100%

A: Control

B: Algal Omega3-DHA 1X treatment (246mg/ kg BW /day)

C: Algal Omega3-DHA 2X treatment (492mg/ kg BW /day)

D: Algal Omega3-DHA + PC 1X treatment (246mg/ kg BW /day)

E: Algal Omega3-DHA + PS 1X treatment (246mg/ kg BW /day)

Table S2. Comparisons of relative organs weight in 6 months-old SAMP8 mice after 13 weeks of algal omega3-DHA treatment.^1, 2^

| Groups | Relative organ weights (g/100g body weight) | | | | | |
| --- | --- | --- | --- | --- | --- | --- |
|  | Brain | Heart | Liver | Spleen | Lung | Kidney |
| **Male** |  |  |  |  |  |  |
| A | 1.46±0.03 | 0.62±0.03 | 4.78±0.12 | 0.26±0.01 | 0.66±0.02 | 1.61±0.02 |
| B | 1.48±0.02 | 0.63±0.03 | 4.97±0.11 | 0.27±0.02 | 0.69±0.02 | 1.64±0.04 |
| C | 1.52±0.02 | 0.67±0.02 | 4.95±0.09 | 0.29±0.02 | 0.67±0.02 | 1.66±0.03 |
| D | 1.50±0.03 | 0.65±0.02 | 4.88±0.10 | 0.27±0.02 | 0.64±0.02 | 1.62±0.03 |
| E | 1.45±0.03 | 0.63±0.02 | 4.80±0.13 | 0.29±0.02 | 0.68±0.02 | 1.63±0.02 |
| **Female** |  |  |  |  |  |  |
| A | 1.48±0.03 | 0.67±0.02 | 4.85±0.08 | 0.27±0.02 | 0.67±0.02 | 1.53±0.02 |
| B | 1.52±0.03 | 0.61±0.02 | 4.80±0.08 | 0.30±0.02 | 0.69±0.03 | 1.55±0.02 |
| C | 1.44±0.04 | 0.66±0.03 | 4.87±0.09 | 0.26±0.03 | 0.67±0.02 | 1.56±0.02 |
| D | 1.47±0.02 | 0.64±0.02 | 4.84±0.09 | 0.25±0.02 | 0.65±0.02 | 1.60±0.02 |
| E | 1.50±0.02 | 0.65±0.03 | 4.81±0.43 | 0.29±0.02 | 0.64±0.02 | 1.57±0.02 |

^1^ Values were expressed as mean ± SEM and analyzed by one-way ANOVA

^2^ Organ weight (%) = (weight of the organ / body weight) *100%

A: Control

B: Algal Omega3-DHA 1X treatment (246mg/ kg BW /day)

C: Algal Omega3-DHA 2X treatment (492mg/ kg BW /day)

D: Algal Omega3-DHA + PC 1X treatment (246mg/ kg BW /day)

E: Algal Omega3-DHA + PS 1X treatment (246mg/ kg BW /day)

Table S3. Hematological parameters of 6 months-old SAMP8 mice after 13 weeks of algal omega3-DHA treatment.^1^

| Groups | A | B | C | D | E |  |
| --- | --- | --- | --- | --- | --- | --- |
| **Male** |  |  |  |  |  |  |
| Glucose (mg/dl) | 115.25 ± 2.90 | 117.42 ± 3.47 | 112.67 ± 3.72 | 114.58 ±2.33 | 111.75 ±2.28 |  |
| Total Protein (g/dl) | 5.30± 0.12 | 5.33 ± 0.14 | 5.38 ± 0.19 | 5.36 ± 0.17 | 5.35 ± 0.19 |  |
| Albumin (g/dl) | 3.10 ± 0.18 | 3.17 ± 0.18 | 3.04 ± 0.23 | 3.01 ± 0.17 | 3.15 ± 0.14 |  |
| Triglyceride (mg/dl) | 105.75 ± 8.03 | 100.50 ± 8.54 | 101.75 ± 8.38 | 100.25 ± 7.84 | 108.58 ± 8.97 |  |
| Total Cholesterol (mg/dl) | 116.17 ± 4.85 | 113.08 ± 6.86 | 107.33 ± 3.23 | 111.50 ± 3.07 | 109.58 ± 2.92 |  |
| HDL (mg/dl) | 55.17 ± 1.84 | 54.58 ± 2.09 | 59.50 ± 3.04 | 55.42 ± 2.25 | 57.67 ± 3.24 |  |
| LDL (mg/dl) | 7.20± 0.23 | 7.17 ± 0.24 | 7.10± 0.16 | 7.13 ± 0.25 | 7.15 ± 0.27 |  |
| AST (U/L) | 84.83 ± 4.50 | 87.50 ± 4.23 | 84.58 ± 5.82 | 85.83 ± 9.34 | 86.92 ± 7.56 |  |
| ALT (U/L) | 59.25 ± 4.33 | 54.83 ± 5.55 | 56.08 ± 5.81 | 57.50 ± 4.03 | 58.25 ± 3.19 |  |
| BUN (mg/dl) | 28.83 ± 1.30 | 27.25 ± 1.94 | 25.25 ± 1.15 | 26.08 ± 1.73 | 26.33 ± 2.19 |  |
| Creatinine (mg/dl) | 0.31 ± 0.03 | 0.29 ± 0.03 | 0.25 ± 0.02 | 0.28 ± 0.02 | 0.26 ± 0.03 |  |
| **Female** |  |  |  |  |  |  |
| Glucose (mg/dl) | | 119.75 ± 2.28 | 116.08 ± 2.77 | 112.08 ± 2.25 | 115.17 ± 2.23 | 113.42 ± 2.51 |
| Total Protein (g/dl) | | 5.35 ± 0.19 | 5.38 ± 0.11 | 5.41 ± 0.20 | 5.33 ± 0.27 | 5.39 ± 0.20 |
| Albumin (g/dl) | | 3.10 ± 0.22 | 3.19 ± 0.22 | 3.08 ± 0.12 | 3.11 ± 0.16 | 3.18 ± 0.16 |
| Triglyceride (mg/dl) | | 103.42 ± 7.76 | 116.00 ± 8.66 | 102.92 ± 9.74 | 109.58 ± 4.97 | 100.58 ± 5.56 |
| Total Cholesterol (mg/dl) | | 118.58 ± 7.03 | 119.00 ± 11.03 | 110.42 ± 7.20 | 116.35 ± 6.32 | 113.83 ± 7.84 |
| HDL (mg/dl) | | 58.17 ± 2.16 | 59.58 ± 2.15 | 52.83 ± 2.80 | 56.50 ± 2.60 | 54.42 ± 2.87 |
| LDL (mg/dl) | | 7.18 ± 0.18 | 7.16 ± 0.19 | 7.12 ± 0.19 | 7.15± 0.16 | 7.13 ± 0.17 |
| AST (U/L) | | 80.75 ± 6.41 | 82.42 ± 5.87 | 85.67 ± 8.86 | 83.33 ± 7.23 | 81.67 ± 6.94 |
| ALT (U/L) | | 58.50 ± 4.01 | 54.42 ± 4.48 | 57.92 ± 4.80 | 56.67 ± 3.59 | 52.42 ± 3.69 |
| BUN (mg/dl) | | 27.58 ± 1.54 | 26.08 ± 1.44 | 23.67 ± 1.38 | 25.33 ± 1.08 | 25.42 ± 1.79 |
| Creatinine (mg/dl) | | 0.32 ± 0.02 | 0.26 ± 0.02 | 0.29 ± 0.02 | 0.27 ± 0.02 | 0.33 ± 0.03 |

^1^ Values were expressed as mean ± SEM and analyzed by one-way ANOVA

A: Control

B: Algal Omega3-DHA 1X treatment (246mg/ kg BW /day)

C: Algal Omega3-DHA 2X treatment (492mg/ kg BW /day)

D: Algal Omega3-DHA + PC 1X treatment (246mg/ kg BW /day)

E: Algal Omega3-DHA + PS 1X treatment (246mg/ kg BW /day)
